# Bilateral type IIpersistent proatlantal intersegmental artery: a rare variant of persistent carotid-vertebrobasilar anastomoses

**DOI:** 10.1259/bjrcr.20210154

**Published:** 2021-12-13

**Authors:** Yajun Fang, Shuhua Li, Chuanchen Zhang

**Affiliations:** 1 Department of MRI, Liaocheng People's Hospital, Liaocheng, Shandong, China

## Abstract

The persistent proatlantal intersegmental artery is a rare variant of persistent carotid-vertebrobasilar anastomoses, especially their bilateral presence is rarer. We report a case of bilateral typeII persistent proatlantal intersegmental artery. The absence of bilateral vertebral arteries was incidentally noted on neck ultrasound examination. Subsequent time-of-flight MR angiography confirmed this. The bilateral typeIIpersistent proatlantal intersegmental artery arose from the cervical external carotid artery, penetrated the C1 transverse foramen, entered the skull via the foramen magnum, and joined the lower portion of the basilar artery.

## Introduction

Persistent carotid-vertebrobasilar artery anastomoses are rare variants and are usually found incidentally. From cranial to caudal position, there is the persistent trigeminal artery (PTA), persistent otic artery (POA), persistent hypoglossal artery (PHA), and persistent proatlantal intersegmental artery (PIA).^1,2^ The most common is the PTA, with an incidence of approximately 0.1–0.6%.^3^ The second most common is the PHA, with an incidence ranging from 0.027 to 0.29%.^4,5^ The PIA and POA are extremely rare. We report a case of bilateral type II PIA found incidentally on head-neck MR angiography examination imaging.

## Case presentation

**Figure 1.**
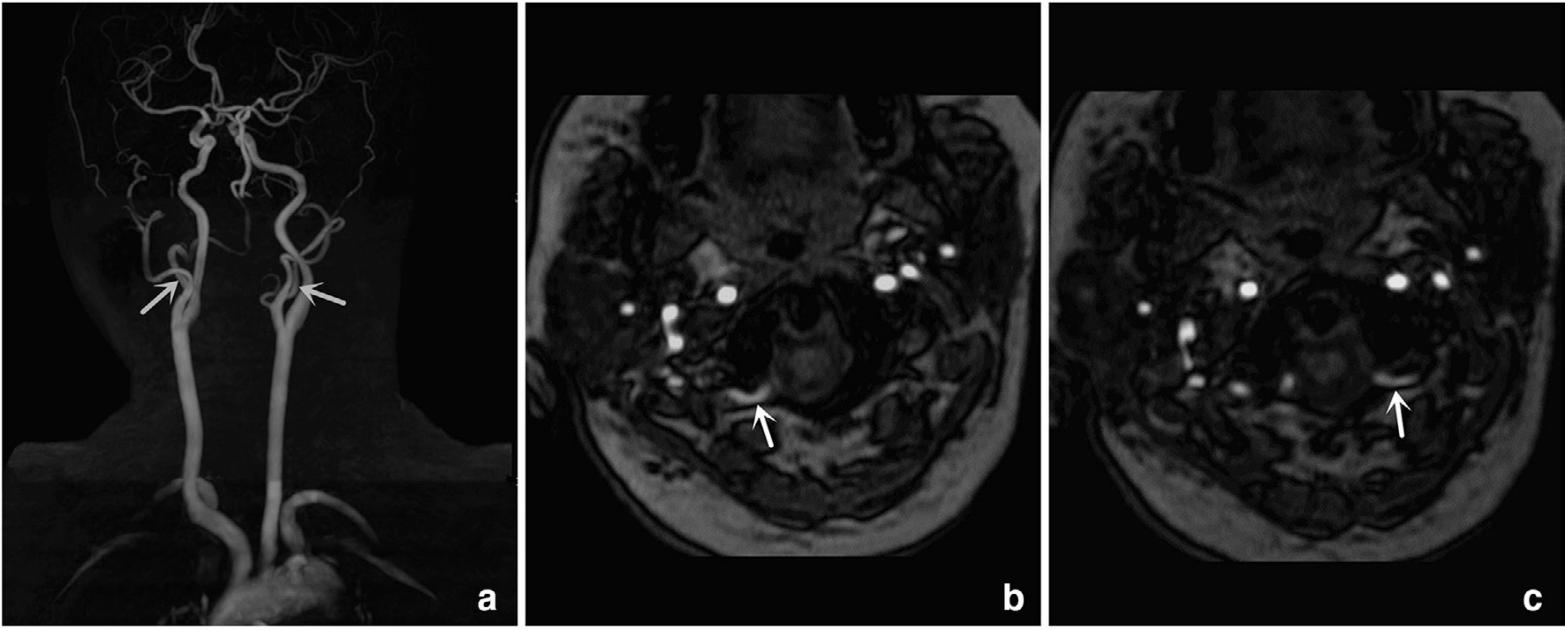
A 47-year-old female with a bilateral type II PIA. (**A**) Coronal: PIA arises from the proximal bilateral ECA (arrow). (**B–C**) Axial: PIA penetrates the C1 transverse foramen, enters the skull via the foramen magnum (arrow)

A 47-year-old female with vertigo underwent neck ultrasound examination, which revealed the absence of bilateral vertebral arteries. Then, she underwent a head-neck time-of-flight (TOF) MR angiography examination, which revealed that the bilateral type IIPIA arose from the bilateral cervical external carotid artery (ECA), traveled between the C1-2 interspace to form V4 segment of the vertebral artery, penetrated the C1 transverse foramen, and entered the skull via the foramen magnum (Figure 1A–C). No abnormality was found in other head-neck arteries. No infarction or other abnormality was noted in the brain.

## Discussion

**Figure 2.**
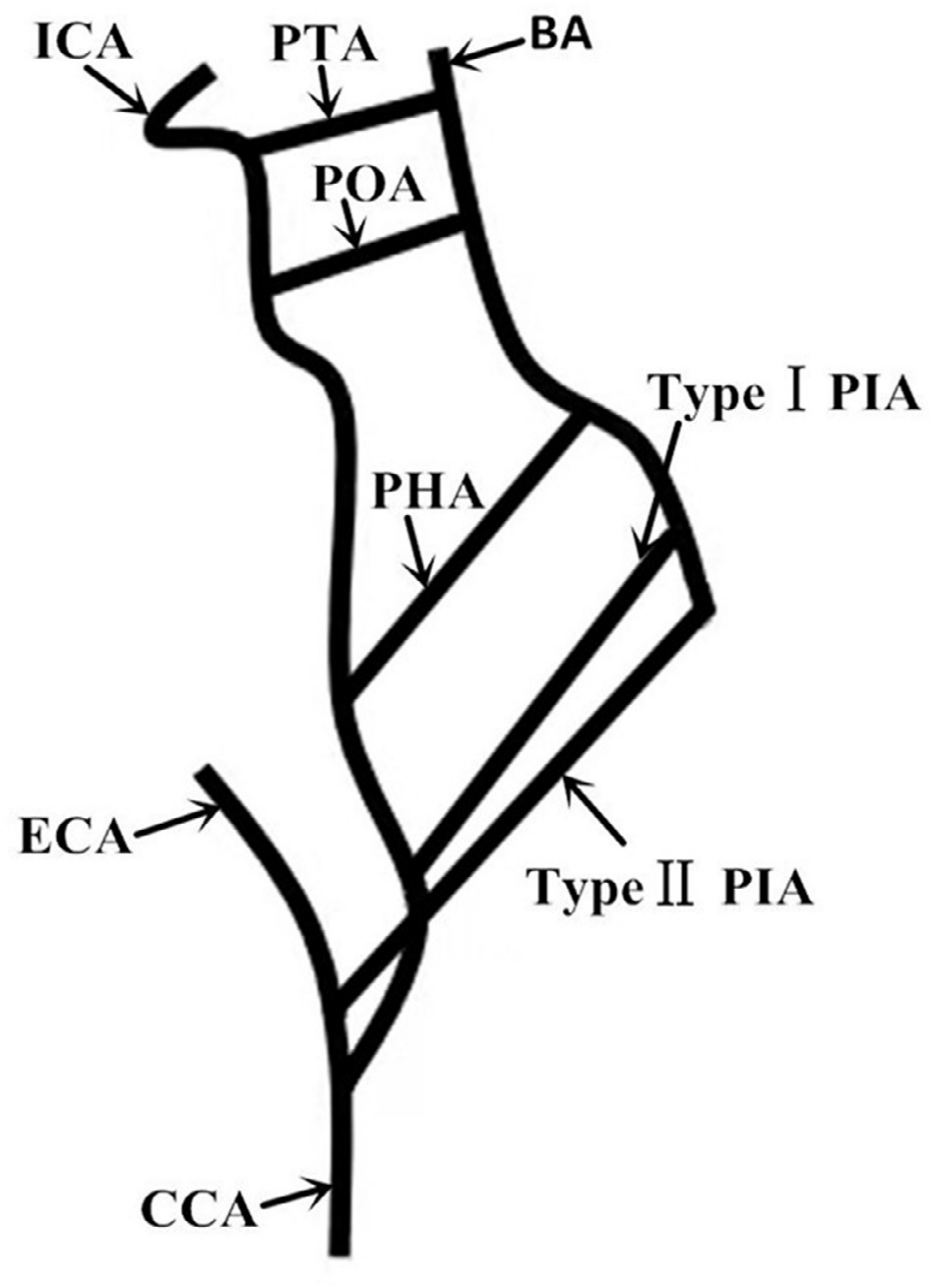
Schematic illustration of carotid-vertebrobasilar artery anastomoses: PTA, POA, PHA, PIA (arrows)

During the 4 to 5 mm embryonic stage, there are four temporary anastomotic arteries: PTA, POA, PTA and PIA from cranial to caudal position, which is named based on their adjacent structures: the trigeminal ganglion, otic vesicle, hypoglossal nerve and proatlantal intersegmental artery.^6^ The major supplier to the cranial and caudal hindbrain is from the trigeminal and proatlantal intersegmental artery, respectively.^3^ During the 7 to 12 mm embryonic stage, the bilateral longitudinal neural arteries later fuse to form the vertebrobasilar system, and the anastomotic arteries begin to regress. The first to regress is the otic artery, followed by the hypoglossal and trigeminal arteries.^3^ The proatlantal intersegmental artery maintains the posterior circulation until the development of the vertebrobasilar system is completed.^7^ Failure of this obliteration results in the persistence of embryonic arteries (Figure 2) and leads to hypoplasia of the vertebrobasilar system.^2^

The PIA is a rare type of persistent carotid-vertebrobasilar artery anastomoses. The PIA originates from the common carotid artery (CCA) bifurcation, ECA or internal carotid artery (ICA) at the C2-4 cervical levels.^3^ Two types are classified. The type I PIA arises from the proximal ICA and courses between the C1 arch and the occiput to join the V3 segment of the VA and enters the posterior fossa via the foramen magnum.^8^ The type II PIA arises from the proximal ECA and travels between the C1-2 interspace to join the V4 segment of the VA, penetrates the C1 transverse foramen, and enters the foramen magnum.^9^ The position of the typeⅡ PIA was lower than that of the type I PIA.^1,10^ Therefore, the type II PIA is usually more tortuous and complex than the type I PIA.^11^ A case of variant PIA was reported in the literature, it originated from ECA, but directly connected with the VA and entered the skull through the foramen magnum. Its origin was the type Ⅱ and its travel was the type I.^12^ The incidence of the left PIA and the right PIA was about 2:1, the bilateral PIA is extremely rare.^13^ In this report, the bilateral type II PIA originated from the bilateral ECA, which was rarely reported in the previous literature.

The development of the PIA usually coincides with the development of the carotid artery and vertebrobasilar artery; it is easy to be associated with vertebrobasilar artery dysplasia or absence.^14,15^ Therefore, it is commonly associated with paroxysmal vertigo, disturbance of consciousness or cross-paralysis and other symptoms of ischaemic cerebrovascular diseases.^16^ The PIA may be accompanied by other cerebrovascular abnormalities or malformations, such as hypoplasia of ICA and ECA, internal carotid-cavernous fistula, VA hypoplasia, intracranial arteriovenous malformation, intracranial aneurysm. Choudhary G analysed 21 cases of the type II PIA and found that most of them were complicated with cerebral vascular abnormalities and two cases were accompanied by aneurysms 1. When the PIA is accompanied by ipsilateral VA absence, the PIA as the main communicating vessel ensures the essential blood supply of posterior circulation. If the PIA is accompanied by severe stenosis or the absence of the ipsilateral ICA, the PIA can be used as a collateral vessel connecting anterior and posterior circulation. When interventional physicians perform endarterectomy for the PIA patients with severe stenosis of the ICA, they need to pay attention to the possibility that the unstable plaque of the ICA may flow into the posterior circulation area through the PIA, which may lead to cerebral infarction in the cerebellum and brain stem.^17^ Li TH reported that the PIA was associated with pulsatile tinnitus, the audible murmur may be due to the increase of the PIA blood flow or turbulence.^18^

The type I PIA is often confused with the variant PHA. They all originate from the cervical ECA. However, they are some differences. Firstly, the original location of the type II PIA is lower than that of the variant PHA. Secondly, the variant PHA has a longer vertical ascending distance than the type II PIA. Most importantly, the type II PIA enters the skull via the foramen magnum, while the variant PHA enters the skull through the enlarged hypoglossal canal.

## Conclusion

The PIA is commonly an incidental finding, however, it can be used as a collateral vessel connecting anterior and posterior circulation. In our case of the bilateral type II PIA, the absence of bilateral vertebral arteries was noted on neck ultrasound examination. TOF MRA can demonstrate the vascular morphology, anatomical characteristics, classification of the PIA. Thus, it may provide a reliable morphological basis for the determination and evaluation of surgical programs, and reduce the operational risks.

## Learning points

1. The PIA is a rare type of persistent carotid-vertebrobasilar artery anastomoses, especially their bilateral presence is rarer. Two types are classified. The type I PIA arises from the proximal ICA and type II PIA arises from the proximal ECA.
2. TOF MRA can demonstrate the vascular morphology, anatomical characteristics, classification of the PIA, which may provide a reliable morphological basis for the determination and evaluation of surgical programs, and reduce the operational risks.
